# Implementation in Nursing Homes: Describing Early and Late Adopters of an Evidence-Based Dementia Care Program

**DOI:** 10.1093/geroni/igab046.2102

**Published:** 2021-12-17

**Authors:** Cara Lekovitch, Pamela Toto, Felicia Chew, Dawn Bieber, Natalie Leland

**Affiliations:** 1 University of Pittsburgh, Pittsburgh, Pennsylvania, United States; 2 Thomas Jefferson University, Mount Royal , New Jersey, United States; 3 Genesis HealthCare, Kennett Square, Pennsylvania, United States

## Abstract

Despite national efforts to improve nursing home (NH) quality, care remains variable. Health system efforts to drive improvement often begin with a sub-group of NHs before scaling up across the organization. Yet, there is limited evidence on who to target for the first group. This study addressed this gap by examining facility characteristics of early and late adopters within a multi-site pragmatic clinical trial. Data were obtained from the Organizational Readiness to Change Assessment (ORCA), which was completed by expert trainers, and Nursing Home Compare. Early and late adoption was operationalized according to Roger’s Diffusion of Innovations. Sixty-percent of NHs (n=12) were late adopters and 40% (n=8) were early adopters. Between group differences (p<.01) were found in number of health inspection citations and context domain within the ORCA. These findings equip health systems with evidence on how to strategically target partners for initial quality improvement efforts prior to system-wide implementation.

